# Attitudes Toward Sexual and Digital Consent and Institutional Distrust as Determinants of Gender-Based Violence Prevention: Evidence from an Urban Adult Population

**DOI:** 10.3390/ijerph23040480

**Published:** 2026-04-10

**Authors:** Esperanza García Uceda, Diana Valero Errazu, Jesús C. Aguerri

**Affiliations:** 1 Department of Management and Organisation, School of Social and Labour Sciences, University of Zaragoza 50009 Zaragoza, Spain; mariola@unizar.es; 2 Department of Psicology and Sociology, School of Social and Labour Sciences, Universidad de Zaragoza, 50009 Zaragoza, Spain; jaguerri@unizar.es

**Keywords:** gender-based violence, sexual violence, sexual consent, digital sexual consent, institutional trust, public health campaigns, primary prevention, Spain

## Abstract

**Highlights:**

**Public health relevance—How does this work relate to a public health issue?**

- Gender-based and sexual violence are major public health concerns, and this study addresses an important preventive dimension: attitudes toward sexual and digital consent in an adult population.
- The study is particularly relevant to contemporary public health because the digitalization of intimate relationships has expanded the risks of consent violation and requires primary prevention strategies to be adapted accordingly.

**Public health significance—Why is this work of significance to public health?**

- The findings show that attitudes toward offline sexual consent and digital consent are closely related, supporting an integrated prevention approach rather than treating these domains separately.
- The study also identifies two key factors for public health prevention: gender, as the most consistent sociodemographic correlate of attitudinal differences, and institutional reluctance, which is systematically associated with less supportive attitudes toward consent.

**Public health implications—What are the key implications or messages for practitioners, policy makers and/or researchers in public health?**

- The results suggest that prevention policies and campaigns should explicitly incorporate digital consent into affective-sexual education and primary prevention strategies for sexual violence.
- The findings also suggest that institutional interventions may benefit from more participatory approaches and greater attention to how messages are received, avoiding moralizing tones and strengthening public trust in order to enhance preventive effectiveness.

**Abstract:**

Gender-based and sexual violence are major public health concerns, and norms about consent are central to their prevention. This study examines how attitudes toward sexual consent relate to digital sexual consent and to the occasional feeling of distrust in public consent campaigns and institutions. We conducted a cross-sectional online survey embedded in the evaluation of a municipal consent campaign in Zaragoza (Spain). Adults (*N* = 404; 56.7% women) completed a 14-item short version of the Sexual Consent Scale–Revised, two items on digital sexual consent, and three items on institutional reluctance (perceived “sermonizing” tone, distrust in effectiveness, and lack of personal identification with the message). Correlation and multiple regression models with robust standard errors were estimated, controlling for gender, age, education, income, relationship status, and social media use. Attitudes toward sexual consent were strongly and positively associated with digital sexual consent. Gender was the most consistent sociodemographic correlate: men showed less egalitarian attitudes than women across all consent measurements. Institutional reluctance was systematically related to less supportive consent attitudes: perceiving institutional messages as exaggerated or personally irrelevant predicted lower support for sexual and digital consent norms, whereas trust in the campaign’s effectiveness was associated with more egalitarian attitudes. The findings support the continuity between sexual and digital consent and highlight gender and institutional trust as key determinants for the prevention of gender-based and sexual violence. Public health and social policies should integrate digital consent into consent education and co-design campaigns that minimize defensive reactions and rebuild trust in institutions.

## 1. Introduction

Gender-based violence, and sexual violence in particular, is now recognized as one of the main public health problems worldwide, due to its high prevalence, the severity of its consequences, and its roots in structural gender inequalities [1,2]. From this public health perspective, sexual violence cannot be addressed solely as an individual or criminal issue but as a socially produced phenomenon requiring comprehensive primary, secondary, and tertiary prevention strategies that align with equality and human rights policies. Within this framework, sexual consent has become a growing priority in public equality policies and in strategies to prevent sexual violence. Primary prevention focuses on intervening before violence occurs, modifying risk factors, and promoting protective factors across multiple levels [3]. Rather than limiting efforts to responses after harm has been done, primary prevention seeks to transform social norms, gender relations, and institutional contexts that allow or tolerate violence [4]. In this context, consent campaigns are conceived as key tools to promote egalitarian norms and communication skills that contribute to reducing the incidence of sexual violence [5,6].

While the importance of consent campaigns is well established, this study addresses a less explored dimension by examining variability in individual responses to institutional consent messages. The effectiveness of public campaigns is influenced by factors beyond the normative content of the message. Research on preventive communication and trust in institutions demonstrates that the reception of a campaign is shaped by processes such as reactance, perceived credibility, and the legitimacy of the issuing institutions [7,8,9,10]. Within this context, the article introduces the concept of institutional reluctance, which remains theoretically underdeveloped in Spanish-language scholarship. Institutional reluctance refers to the distrust or critical distance that certain groups maintain toward official consent messages, particularly when campaigns are perceived as overly didactic or disconnected from real-life experiences. In this study, the concept is operationalized specifically in relation to public municipal campaigns on consent—not to institutional trust in a broader sense (e.g., toward educational, judicial, or healthcare institutions). It captures three distinct attitudinal stances: perceiving campaign messaging as moralizing or exaggerated in tone, doubting the effectiveness of campaigns in preventing assaults, and failing to personally identify with the messages. These dimensions are treated as separate variables rather than a composite scale, given the empirical absence of a common underlying factor (see Section 3.2).

This dynamic creates a paradox: negative reception of institutional messages may correlate with less egalitarian attitudes toward consent among the very groups targeted by these campaigns. Scholarship on the unintended effects of campaigns describes such responses as “boomerang effects,” where preventive communication inadvertently reinforces the beliefs or practices it aims to change [8,11]. The present study has three interconnected aims: (1) to examine the relationship between attitudes toward offline and digital sexual consent in a community adult sample; (2) to identify the sociodemographic factors—particularly gender—associated with both dimensions; and (3) to assess whether different forms of institutional reluctance toward public consent campaigns are associated with less supportive consent attitudes. The study does not seek to establish causal relationships regarding behavior. Rather, it maps attitudinal associations to better understand why some individuals are less receptive to institutional prevention messages, situating this analysis within a primary prevention framework for gender-based and sexual violence.

At the same time, the digital transformation of intimate and sexual interactions has introduced new dimensions of vulnerability and normative complexity. The exchange of sexual content through social media and messaging applications, the unauthorized circulation of intimate images, and the technical ease with which private material can be shared without consent represent relatively recent phenomena that require adapting traditional conceptual frameworks on sexual consent [12,13,14]. Research on technology-facilitated sexual violence has also shown that these practices intersect with inequalities related to gender, age, and sexual orientation, thereby amplifying the impact of digital abuse on specific groups [15,16].

In this context, digital consent presents distinctive features that partially differentiate it from consent in face-to-face interactions: the permanence of content once disseminated and the difficulty of fully retrieving or deleting images; a potentially uncontrollable reach, given the porous boundaries between private and public online spaces; the practical irreversibility of the decision to share, which makes it very difficult to revoke consent afterwards; and the emergence of new forms of abuse, such as the non-consensual distribution of intimate images, so-called revenge porn, or the use of sexual deepfakes [12,17,18]. These specific features situate digital consent at the intersection of sexual ethics, digital rights, and data protection. They also pose particular challenges for public prevention policies.

Recent literature suggests that attitudes toward consent in offline and digital contexts are closely related. However, the specific mechanisms underlying this relationship—especially when considering factors such as trust or distrust in institutions—remain insufficiently explored [14,19,20]. One of the methodological contributions of this study is the integration of attitudes toward sexual consent, attitudes toward digital consent, and different forms of institutional reluctance into a single empirical model for the first time in the Spanish context. This approach enables analysis of how these dimensions interact and how the perception of institutional messages modulates the internalization of egalitarian norms in both offline and digital environments.

The central hypothesis guiding this study is that institutional reluctance—understood as the perception that institutional consent messages are exaggerated, ineffective, or disconnected from one’s own reality—is associated with less supportive attitudes toward sexual and digital consent norms. Specifically, this manifests as lower endorsement of the need to establish explicit consent, reduced recognition of consent as a shared responsibility, and greater tolerance for ambiguous or assumed consent. By “less egalitarian,” we refer to attitudinal positions that are less aligned with frameworks of affirmative, ongoing, and mutual consent, as operationalized in the present study through the SCS-R dimensions and the digital consent index. In other words, the argument is that how individuals perceive and evaluate consent campaigns may modulate their internalization of consent norms. Given that the available data concern attitudes and self-assessments, these relationships should be interpreted as associations rather than evidence of direct causal effects on behavior.

This perspective carries significant implications for public policy design. If institutional reluctance functions as a mechanism that influences campaign effectiveness, then increasing the volume or refining the normative content of messages is not enough. It is essential to explicitly address how target groups perceive the credibility, relevance, and authenticity of these messages, as well as the alignment between institutional messaging and actual institutional practices [10,16]. The empirical analysis of these dynamics represents the principal contribution of this article to the discourse on sexual and digital consent within primary prevention.

## 2. Theoretical Framework

In recent decades, sexual consent has evolved from being conceived as a simple “one-time act” of acceptance or refusal to being understood as a relational, contextual, and multidimensional process. Much of the literature agrees that consent involves at least three interrelated levels: (a) the internal willingness to participate in sexual activity, (b) the way in which that willingness is (or is not) communicated to the other person, and (c) the social and gender norms that shape how such signals are interpreted. Authors such as Beres [21,22] have shown that although policies and campaigns have evolved from the slogan “no means no” to messages such as “obtain consent” or “only yes means yes,” the everyday experience of consent among young people and adults remains far more complex, marked by ambivalence, contradictory expectations, and unequal power contexts.

A central axis of this debate is the distinction between internal and external consent. Following the classic definition by Hickman and Muehlenhard [23], consent can be understood as the freely made decision to participate in a specific sexual interaction—a decision that may align, to varying degrees, with what the person feels internally and ultimately expresses. Subsequent studies have specified that internal consent refers to feelings of desire, comfort, safety, agreement, and readiness, whereas external refers to the range of verbal and nonverbal signals—ranging from an explicit “yes” to gestures, bodily responses, or the “continuity” of the interaction—through which willingness is communicated [6,24,25].

This distinction helps explain why the mere presence of a positive external signal does not, by itself, guarantee ethical or desirable consent. Research on discrepancies between internal and external consent shows that individuals may “go along with” or continue a sexual encounter despite a lack of genuine willingness—due to partner pressure, fear of social consequences, or gender scripts that discourage explicit refusal [22,26,27]. These studies emphasize that the goal of prevention cannot be limited to ensuring a minimal form of external consent; rather, it must aim to align, as far as possible, desire, comfort, and effective decision-making ability.

Beyond this intrapersonal and communicative layer, several authors highlight that consent is shaped by social norms and gendered scripts that define which behaviors are perceived as “expected” or “acceptable” for men and women. From the perspective of sexual script theory [28], consent is embedded in cultural narratives that typically assign men the initiative and active pursuit of sex, and women the responsibility of regulating access and managing risk. At times, women are also expected to “soften” rejection. Recent studies synthesized by Anyadike-Danes et al. (2024) [29] and Knountsen et al. (2024) [30] show that implicit expectations that foster ambiguity persist, even in contexts where egalitarian discourses are predominant. These expectations are particularly prevalent when attraction or certain initial gestures are understood as implying an “obligation” to continue the interaction. These normative and structural dimensions are essential for interpreting attitudes toward consent and, in our case, for understanding why gender emerges as a central axis in the differences observed.

### 2.1. Sexual Consent as a Multidimensional Construct

Various frameworks have proposed moving from minimal models (“no means no”) to more robust conceptions such as affirmative or enthusiastic consent, which underscore the need for active, ongoing, and clearly voluntary participation by all parties involved [22,31]. These approaches understand consent as a dynamic, reversible, and context-sensitive process that must be sustained throughout the sexual interaction and not only at its outset. From a public policy perspective, this conceptual shift has direct implications: campaigns limited to simplified slogans may be insufficient—or even counterproductive—if they do not help develop the skills to ask, listen, negotiate, and respect changes in consent as the encounter unfolds.

Recent research on internal and external sexual consent has reinforced this multidimensional view by incorporating specific scales designed to capture feelings, cognitions, and behaviors related to consent (e.g., the Internal Consent Scale and related instruments [20,24,26]). The Sexual Consent Scale–Revised (SCS-R) has become one of the most influential instruments for assessing beliefs, attitudes, and behaviors related to the negotiation of sexual consent [32]. The SCS-R was explicitly developed drawing on the Theory of Planned Behavior [33] and organizes the construct into several factors. Two dimensions in particular stand out as especially relevant for explaining both offline and digital consent: perceived (lack of) behavioral control and positive attitudes toward establishing consent. Later studies have confirmed the scale’s solid reliability and validity in different contexts, emphasizing its usefulness in linking consent to other phenomena such as rape-supportive attitudes [32,34].

In recent years, adaptations and validations of the SCS-R have multiplied across countries, enabling the analysis of consent in more diverse community samples. For example, Rollero et al. [34] found that lower levels of sexual consent are associated with more favorable attitudes toward rape in a large heterosexual adult sample. This highlights the role of consent as a protective factor against sexual violence. In Spanish-speaking contexts, the adaptation of the SCS-R and related scales has confirmed the construct’s multifactorial structure and the importance of dimensions related to behavioral control and consent norms [26].

However, the authors themselves stress that these tools should be understood as partial operationalizations of a broader phenomenon that includes affective (desire, comfort, safety), cognitive (beliefs and expectations about what consent “should” be), behavioral (ways of requesting and expressing consent), and structural dimensions (gender norms, power inequalities, histories of violence). In this study, the SCS-R is primarily used as an approximation to these components, but it is situated within a theoretical conception of consent that incorporates these multiple layers.

Alongside this psychometric approach, an influential body of literature insists that consent should be understood not only as a legal or formal marker, but also as a relational and situated practice. Beres [22] showed that many young people conceptualize consent as a minimum requirement for sex to be “acceptable,” yet describe in much more complex terms how they negotiate desire, doubt, or refusal in their everyday relationships. This distinction between “internal consent” (truly feeling willing) and “external consent” (what is communicated verbally or nonverbally) has been revisited by recent studies that analyze how discrepancies between the two have important implications for well-being and sexual satisfaction [6,26,35].

Contemporary empirical work has further demonstrated that internal and external consent are not static attributes; rather, they vary substantially within individuals across situations and encounters, depending on contextual and relational factors [35]. Moreover, situational conditions such as alcohol or cannabis use have been shown to increase discrepancies between internal willingness and external expressions of consent. This reinforces the need to conceptualize consent as a dynamic and context-dependent process rather than a fixed decision [25].

Research has also shown that the effectiveness of affirmative consent initiatives is constrained not only by individual attitudes but also by broader social, relational, and institutional factors. Using the Social Ecological Model, Willis and Jozkowski [36] demonstrate that barriers to affirmative consent operate at multiple levels, including interpersonal dynamics, peer norms, and institutional contexts, thereby limiting the impact of campaigns based solely on normative messaging.

### 2.2. Gendered Sexual Scripts and Differences in Attitudes Toward Consent

One of the most consistent findings in the literature is the existence of gender differences in attitudes toward consent and in how sexual practices are negotiated [35]. These variations are commonly interpreted through the lens of sexual script theory, which conceives sexuality as organized by cultural narratives that define which behaviors are expected of men and women [28]. According to this theory, men are usually socialized as the initiators of sexual contact, with greater agency and legitimacy to propose, while women are socialized as the “gatekeepers” of sexuality, with a role more focused on regulating boundaries and protecting themselves from risk [28,35].

These scripts have direct implications for consent. Recent studies show that women generally express stronger support for egalitarian norms and the need for explicit consent, as well as greater sensitivity to its absence across different contexts [26,35]. At the same time, they may experience lower levels of perceived behavioral control, feeling less legitimized to verbalize desires or refusals in situations that contradict these traditional scripts [26]. Men, conversely, tend to show greater confidence in their ability to negotiate sexual encounters, but also a higher likelihood of relying on indirect signals or assumptions about consent grounded in gender expectations [34,35].

Research focusing on explicit verbal consent communication further indicates that gender differences are shaped by relational context and sexual precedent. Willis and Jozkowski [37] showed that prior sexual interactions often reduce the likelihood of explicit consent communication, particularly in established relationships, where assumptions about willingness may replace renewed consent. Similarly, studies examining different types of sexual behavior found that explicit verbal consent is more common in casual or incipient relationships and less frequent in ongoing partnerships, especially among men [6].

It is equally important to acknowledge that developments in communication technologies have given rise to new sexual practices mediated by digital devices and platforms, prompting a reformulation of consent beyond face-to-face encounters [38]. Digital consent can be defined as the need to obtain explicit permission before producing, sending, or sharing intimate or sexual content involving another person in online environments [39]. This notion incorporates elements such as the potential permanence of content, the ease of uncontrolled dissemination, the difficulty of revoking consent once material has been shared, and the emergence of new forms of violence, such as the non-consensual distribution of intimate images or revenge pornography [39,40].

Recent literature suggests a meaningful continuity between attitudes toward sexual consent in offline contexts and those toward digital consent [35]. Individuals who value explicit communication in sexual encounters also tend to consider consent indispensable before sending or forwarding sexual content via social media or messaging applications [35,39]. Extending this perspective, Willis and Smith [20] show that consent-related attitudes vary across behaviors and contexts, and that gender differences and prior nonconsensual sexual experiences play a key role in shaping how consent is interpreted and enacted, reinforcing the view of it as a cross-contextual ethical framework rather than a context-specific rule. However, the digital environment—and the sometimes misleading sense of privacy it creates—introduces more ambiguity into digital consent than into in-person, as the perception of crossing consent boundaries tends to be lower.

### 2.3. Institutional Reluctance and Distrust Toward Public Messages

Evaluations of prevention campaigns show heterogeneous results: some produce short-term improvements in knowledge and certain attitudes, but their sustainability and ability to modify behaviors are more limited [5,41]. Among the factors influencing their effectiveness, the literature highlights psychological reactance, perceived credibility, and institutional legitimacy [7,8,10]. The most effective approaches are those that address the complexity of consent, incorporate the participation of target groups, and are embedded in broader strategies of community and institutional change [3].

Here, institutional reluctance refers to the critical distance or skepticism that certain segments of the population display toward messages issued by public institutions on sensitive topics such as consent or sexual violence [42]. This reticence may be expressed in several ways: perceiving campaigns as exaggerated or moralizing, distrusting their ability to prevent assaults, or failing to personally identify with messages that feel disconnected from one’s own reality. While these dimensions do not constitute a single factor, they may coexist and reinforce one another.

Research on institutional trust has shown that the gap between official messages and the everyday experiences of the public is a key predictor of distrust and resistance to public policies [42,43]. When messages are perceived as top-down, overly simplified, or poorly connected to the lived realities of target groups, the likelihood of rejection or indifference increases [42]. In the field of sexual health and violence prevention, this distrust can translate into reduced willingness to change attitudes or behaviors, even when the content of campaigns is technically sound [5].

### 2.4. The Theory of Planned Behavior as an Integrative Framework

The Theory of Planned Behavior (TPB) offers a useful framework for understanding how attitudes, subjective norms, and perceived control combine to predict intentions and behaviors [33,44]. When applied to sexual and digital consent, it facilitates an interpretation of how beliefs about the importance of consent, perceptions of social norms, and feelings of ability influence the likelihood of requesting, granting, or refusing consent in both offline and digital contexts.

In the present study, the variables employed—GASC, SCS-F1, SCS-F2, and digital consent—align with the three components of TPB. Institutional reluctance introduces an additional contextual element that may shape the processes through which campaigns aim to transform attitudes and norms. TPB has been widely used to analyze the effectiveness of public health communication campaigns, violence-prevention strategies, and the promotion of safe behaviors, which reinforces its suitability as an integrative framework for this study.

The theoretical framework presented here articulates four central ideas:

(1) Sexual consent is a multidimensional construct that includes cognitive, affective, and behavioral components and can be measured with consolidated instruments [26,32,34].
(2) While digital consent shares a normative foundation with its in-person counterpart, it also incorporates specific risks linked to the circulation of intimate images and the logic of social media [35,39].
(3) Gender acts as a structural axis that organizes unequal sexual scripts, particularly regarding sexual initiative; interpretation of consent; and risk-taking [28,35].
(4) Trust or distrust in institutions shapes the context in which the population receives and processes preventive messages [42].

Building on this foundation, the present study analyzes how these dimensions combine and manifest in attitudes toward sexual and digital consent, and to what extent institutional reluctance is associated with less egalitarian positions. Specifically, it seeks to investigate the relationship between attitudes toward sexual consent and “consent in the digital world,” to identify sociodemographic factors associated with both dimensions, and to examine the role institutional reluctance—understood as distrust or critical distance toward institutional messages on consent—plays in shaping such attitudes. To achieve this objective, the study draws on the following four research questions.

⋅ RQ1: How are attitudes toward sexual consent related to those toward digital consent?
⋅ RQ2: Which sociodemographic factors are associated with attitudes toward sexual and digital consent?
⋅ RQ3: Which sociodemographic profiles display higher or lower reluctance toward institutional messages on consent?
⋅ RQ4: To what extent does reluctance predict less egalitarian or more permissive attitudes toward the absence of consent?

By addressing these research questions, the study seeks to inform social and equality policies using empirical evidence on attitudes toward consent in digital contexts. It also aims to identify sociodemographic profiles associated with more permissive attitudes toward consent or greater rejection of institutional campaigns, thereby facilitating the adaptation of interventions to groups with lower receptivity.

## 3. Materials and Methods

### 3.1. Study Design and Context

This study is based on secondary data analysis. The dataset was originally collected by the Chair of Social Policies of the University of Zaragoza and Zaragoza City Council, as part of the evaluation of a municipal sexual consent awareness campaign. The research team was granted access to the dataset by the commissioning institution under a data-sharing agreement. The data were provided in fully anonymized form, with no personal identifiers retained, making it impossible to trace any response back to an individual participant. No new data were gathered directly from human participants for the purposes of this study. Under Spanish legislation, ethics committee review applies to research involving direct intervention on human participants or primary collection of personal data (Ley 14/2007, de 3 de julio, de Investigación Biomédica, Art. 2 and 3; Real Decreto 1716/2011, de 18 de noviembre). Secondary analysis of pre-existing, fully anonymized data provided by a public institution does not fall within the scope of mandatory ethics review under this legal framework. Data protection compliance was ensured in accordance with Regulation (EU) 2016/679 of the European Parliament and of the Council (General Data Protection Regulation, GDPR).

The dataset comprises 404 adult participants residing in the city of Zaragoza. The original sample was constructed using a panel-type design stratified by age and gender, and the questionnaire was administered via an online self-report format. Although the study is based on a local sample, the sociodemographic composition of participants suggests that the findings may be cautiously informative beyond the Zaragoza context. The sociodemographic characteristics of the sample—detailed in Table 1—do not suggest major imbalances, although the findings should be interpreted in light of the study’s local and self-reported design.

The data were analyzed using both bivariate tests and robust linear regression models.

### 3.2. Instruments

#### 3.2.1. Attitudes Toward Sexual Consent

To measure attitudes, beliefs, and perceived behaviors related to the negotiation of sexual consent, an abbreviated version of the Sexual Consent Scale–Revised (SCS-R) [32] was employed. The SCS-R is a widely used instrument for assessing consent in the sexual domain [45]. The original version consists of 39 items on a 7-point Likert scale (1 = strongly disagree; 7 = strongly agree). These items are grouped into five subscales or factors: SCS-R F1: (Lack of) perceived behavioral control; SCS-R F2: Positive attitude toward establishing consent; SCS-R F3: Indirect behavioral approach to consent; SCS-R F4: Norms about sexual consent; and SCS-R F5: Awareness and discussion of consent.

Due to practical constraints, the present study used an abbreviated version of the SCS-R proposed and validated by Rollero et al. [34]. This adaptation reduces the number of items to 15 while maintaining the original scale’s five-factor structure. Given the target population, these 15 items were administered in Spanish, using the translation of the SCS-R developed and validated by Moyano et al. [26] (see Table 2). Once the data were collected, a confirmatory factor analysis was performed. The adequacy of the correlation matrix for a factorial model was satisfactory (KMO = 0.74), and Bartlett’s test of sphericity was significant: χ^2^(105) = 1760.52, *p* < 0.001. Cronbach’s alpha for the full 15-item scale was α = 0.73. In the theoretical subscales, reliability was good for F1 (α = 0.77) and F2 (α = 0.75), while F3 (α = 0.60), F4 (α = 0.50), and F5 (α = 0.59) showed more modest values. In addition, an exploratory factor analysis with five factors also showed a loading pattern largely consistent with the original theoretical structure. Overall, communalities for most items were moderate–high (h^2^ ≥ 0.51). The main exception was item q10, which presented the lowest communality in the entire scale (h^2^ = 0.28) and a moderate maximum loading (≈0.40) on its theoretical factor; consequently, this item was removed, resulting in a slight increase in the overall reliability (α = 0.74 in the 14-item version).

A global index of attitudes toward sexual consent (GASC) was calculated from this 14-item scale as the mean of the different item scores. In addition, given their robustness, the first two factors of the scale, i.e., the (Lack of) perceived behavioral control (SCS-F1) and the Positive attitude toward establishing consent (SCS-F2), were also used as independent dependent variables. In the case of SCS-F1, higher scores reflect greater difficulty in requesting, negotiating, or establishing consent—that is, a lower sense of perceived behavioral control.

#### 3.2.2. Digital Sexual Consent

Consent in the digital sphere was assessed using two items designed to capture attitudes toward the importance of consent in behaviors involving sexual content in these environments. Participants were asked whether they considered it important to obtain consent before sending sexual content through social media and before sharing someone else’s intimate images, regardless of the type of prior relationship. Both items used a 1–7 Likert response scale. These two items were developed specifically for this study to capture the most widely discussed forms of digital consent violation in the recent literature—namely, the non-consensual sending of sexual content and the unauthorized sharing of intimate images [12,18]. They were designed to be contextually accessible to a general adult population and to align conceptually with the offline consent dimensions measured by the SCS-R (particularly SCS-F2, positive attitude toward establishing consent). While not drawn from a pre-existing validated scale for digital consent—given the current absence of such instruments validated for Spanish adult community samples—their content validity is grounded in the digital consent literature [14,20], and their reliability was empirically verified prior to analysis. Since both indicators conceptually refer to the same construct, their internal consistency was examined to determine whether they could be aggregated into a single composite indicator. The reliability analysis showed a high correlation between the two items and a Cronbach’s alpha of α = 0.78, indicating good internal consistency. Accordingly, a global index of digital consent was constructed by averaging the two items, with higher scores reflecting greater importance attributed to consent in these contexts.

#### 3.2.3. Reluctance

Reluctance toward institutional messages about consent was assessed using three items designed to capture different dimensions of skepticism or distancing toward such campaigns. The statements included: (r1) “I feel that institutional messages about consent tend to exaggerate or lecture”, (r2) “I trust that institutional campaigns on consent actually help prevent assaults”, and (r3) “Messages about consent in affective or sexual relationships are not for me/do not apply to my reality.” All items were answered on a 1–7 Likert scale. The underlying structure of these three statements was examined through exploratory factor analysis and bivariate correlations. The results showed the absence of a common factor, very low item correlations, and insufficient reliability to consider them a unidimensional scale. This lack of conceptual cohesion suggests that each item captures a distinct form of reluctance—criticism of tone, level of trust in the perceived usefulness of campaigns, and lack of personal identification. For this reason, the three items were used as separate variables in subsequent analyses. This allows us to examine how each specific dimension of reluctance relates differentially to the attitudes under study.

### 3.3. Data Analysis Strategy

The data analysis proceeded in several stages. First, descriptive statistics (means, standard deviations, and frequencies) were calculated for the sociodemographic variables and for the indices of sexual consent, digital consent, and institutional reluctance. Second, the internal reliability and factorial structure of the abbreviated SCS-R were evaluated through exploratory and confirmatory factor analyses, following the procedures described in Section 4.2.

Next, bivariate correlations were estimated among the key variables (the global index of attitudes toward sexual consent, the SCS-F1 and SCS-F2 factors, the digital consent index, and the three reluctance items). Several linear regression models with robust standard errors (HC2) were then constructed to address the research questions. Specifically, the following models were fitted: (a) models analyzing the relationship between sexual (GASC, SCS-F1, SCS-F2) and digital consent, (b) models exploring the association between attitudes toward consent and sociodemographic factors, (c) models examining the sociodemographic profiles associated with each form of institutional reluctance, and (d) models incorporating the three dimensions of reluctance as predictors of attitudes toward sexual and digital consent.

## 4. Results

### 4.1. Attitudes Toward Consent and Digital Consent

The bivariate correlation between the global attitude toward sexual consent (GASC) and the digital consent index (DC) was moderate and statistically significant (r = 0.375, *p* < 0.001), indicating that higher scores on egalitarian attitudes toward sexual consent are associated with greater importance attributed to consent in digital contexts.

Based on this relationship, several linear regression models with robust standard errors (HC2) were estimated using digital consent (DC) as the dependent variable. In an initial model including only GASC as a predictor, the global attitude toward sexual consent showed a positive and considerable effect on digital consent (β ≈ 0.58), explaining approximately 18% of the variance (R^2^ ≈ 0.18). When sociodemographic controls (gender, age, educational level, income, frequency of social media use, and relationship status) were introduced, the coefficient associated with GASC remained stable and statistically significant, indicating that the association between the two forms of consent cannot be attributed solely to sociodemographic differences.

Given the strong psychometric performance of the SCS-F1 and SCS-F2 factors, additional models in which digital consent was predicted from these two specific dimensions were estimated. The strongest effect on digital consent was observed for SCS-F2 (Positive attitude toward establishing consent) (β ≈ 0.73, R^2^ ≈ 0.48): individuals who place greater importance on discussing and agreeing on consent also report greater importance of consent in digital contexts.

By contrast, the SCS-F1 dimension [(Lack of) perceived behavioral control] showed a negative association with digital consent (β ≈ −0.24, R^2^ ≈ 0.12). That is, those who report greater difficulty requesting or negotiating consent in offline sexual settings also tend to consider digital consent less relevant. When both SCS-F1 and SCS-F2 were included in the model simultaneously, SCS-F2 remained the central predictor, while the effect of SCS-F1 decreased, it retained its statistical significance, suggesting that both dimensions capture complementary aspects of attitudes toward consent (see Table 3).

Subsequently, associations between digital consent and the two most robust SCS-R dimensions identified in the factorial analyses were examined:

(1) (Lack of) perceived behavioral control (SCS-F1), and
(2) Positive attitude toward establishing consent (SCS-F2).

The correlations were statistically significant for both factors, although in opposite directions (r = −0.291 for SCS-F1 and r = 0.656 for SCS-F2; *p* < 0.001 in each case). Regarding the (Lack of) perceived behavioral control, the negative relationship indicates that a greater sense of inability to request or establish sexual consent is related to less favorable attitudes toward digital consent. In other words, difficulties perceived in the offline sexual domain appear to transfer to the digital context as well.

In a different vein, the correlation between digital consent and positive attitude toward establishing consent was notably high (r = 0.656), suggesting a strong connection between valuing the explicit establishment of consent and recognizing the need for consent before sending, sharing, or exchanging sexual content in digital environments. Regression models confirmed this pattern. For (Lack of) perceived behavioral control, the model significantly predicted less favorable attitudes toward the importance of digital consent (β = −0.254, SE = 0.043, *p* < 0.001), explaining 8.5% of the variance. This effect persisted when sociodemographic controls were added (β = −0.235, *p* < 0.001; R^2^ = 0.122). For a Positive attitude toward establishing consent, the effect was even stronger: β = 0.740 (SE = 0.047, *p* < 0.001) without controls and β = 0.732 (SE = 0.048, *p* < 0.001) with controls, showing a substantially higher explained variance (R^2^ = 0.430 and 0.479, respectively). None of the sociodemographic variables altered the magnitude of the SCS-F1 or SCS-F2 effects, which remained the central predictors.

Overall, the results show that attitudes toward sexual consent are clearly and strongly related to attitudes toward digital consent, even when controlling for gender, age, educational level, income, social media use, and relationship status. This association is particularly strong for the latter dimension reflecting a positive attitude toward establishing consent (SCS-F2). This indicates that those who value explicit communication of consent in offline contexts also tend to recognize its importance in digital exchanges. Conversely, perceived difficulties in managing consent (SCS-F1) are associated with less favorable attitudes toward digital consent.

### 4.2. Sociodemographic Factors Associated with Attitudes Toward Consent

To identify sociodemographic factors linked to attitudes toward consent in both physical and digital contexts, four regression models were constructed:

(1) A model with the global index of attitudes toward sexual consent (GASC);

(2) A model with the digital consent index; and

(3–4) Two additional models analyzing separately the two most robust dimensions identified in the psychometric analyses of the SCS-R: SCS-F1 (Lack of perceived behavioral control) and SCS-F2 (Positive attitude toward establishing consent).

All four models included the same sociodemographic predictors: gender, age, educational level, frequency of social media use, income, and relationship status (see Table 4).

The results show that gender is the only sociodemographic predictor with clear, consistent, and statistically significant effects across all dimensions analyzed, although with some nuances in each case.

The model using GASC as the dependent variable indicates that women display more favorable attitudes toward sexual consent than men (β = 0.240, *p* < 0.05). The remaining variables—age, income, social media use, and relationship status—do not show significant effects, while education exhibits a positive but non-significant trend (β = 0.035). This model explains 3% of the variance (R^2^ = 0.030), suggesting that although gender has a clear effect, attitudes toward sexual consent are also shaped by psychological and attitudinal factors not included among these predictors.

For digital consent, the pattern is similar but slightly more pronounced. Again, gender emerges as a significant predictor with an even larger effect than in the previous model. Women tend to place greater importance on consent in digital contexts, as mentioned in the previous paragraph, (β = 0.458, *p* < 0.01). Educational level is also a significant predictor (β = 0.118, *p* < 0.05), although its effect is notably small. The model’s explained variance remains low at 5.1% (R^2^ = 0.051).

Gender also emerged as a significant predictor for both (Lack of) perceived behavioral control and Positive attitude toward establishing consent. Specifically, for (Lack of) perceived behavioral control, the effect is significant and negative (β = −0.531, *p* < 0.01), indicating that women report lower perceived lack of control—that is, they feel more confident and capable of establishing sexual consent. None of the remaining predictors showed a significant effect in this model. For a Positive attitude toward establishing consent, both gender (β = 0.417, *p* < 0.01) and educational level (β = 0.112, *p* < 0.05) were significant, although the effect of education remained very small again.

Thus, in relation to RQ2—which sociodemographic factors are associated with attitudes toward sexual and digital consent—our data indicate that gender is the only sociodemographic factor with clear, robust, and consistent effects across all analyzed dimensions. Women displayed more favorable attitudes toward the importance of sexual consent, greater confidence and perceived control in their consent-related behavior, and a more positive evaluation of the importance of digital consent. Compared to men, they tended to exhibit more favorable attitudes toward sexual consent (higher GASC); lower perceived behavioral control difficulties (SCS-F1), meaning they feel more capable of establishing consent; more positive attitudes toward explicit consent (SCS-F2); and a greater emphasis on digital consent. These gender effects persisted even after controlling for all other sociodemographic variables.

The remaining predictors, however, showed weaker and inconsistent associations. The effects of age were only occasional and very small, without a clear or stable pattern across models. Educational level tended to be associated with slightly more egalitarian attitudes toward consent (higher GASC and SCS-F2) and with a small reduction in perceived difficulties (lower SCS-F1), but coefficients are moderate and not consistently significant. Income, social media use, and relationship status exhibit very modest or null effects on attitudes toward sexual and digital consent, without forming clearly differentiated profiles.

### 4.3. Reluctance and Associated Factors

As noted earlier, it was not possible to construct a global index of reluctance, so each item used to measure it was analyzed separately: criticism of campaign tone (r1), level of trust in the effectiveness of campaigns (r2), and lack of personal identification with the messages (r3). Models using each item as a dependent variable allow exploration of which sociodemographic profiles are associated with greater skepticism toward institutional messages.

The clearest results emerged for the item referring to the tone of institutional consent campaigns (r1). This was the only item for which a significant predictor was identified: gender. Women were significantly less likely than men to agree with the statement (β = −0.768, *p* < 0.001). This indicates that men tend to perceive institutional campaigns as exaggerated or moralizing. Age was also a significant predictor, albeit with a very small effect (β = −0.018, *p* < 0.05). No other variables showed significant effects. This model explains 7.6% of the variance (R^2^ = 0.076), the highest among the three items, although still moderate in absolute terms.

For the remaining models, none of the sociodemographic predictors was statistically significant, and the explained variance was extremely low. Consequently, it was not possible to identify sociodemographic factors associated with either trust in the impact of institutional campaigns (r2) or lack of personal identification with institutional messages (r3).

The analysis of the relationship between the reluctance items and attitudes toward consent did yield more substantial results. As shown in Table 5, the three reluctance items (r1, r2, and r3) were differentially associated with less egalitarian attitudes toward consent at various levels.

For GASC, two forms of reluctance showed clear and significant negative effects. The perception that campaigns exaggerate or adopt a moralizing tone (r1) predicted less favorable global attitudes toward the importance of sexual consent (β = −0.164, *p* < 0.001). The lack of identification with the messages (r3) was also significantly associated with less egalitarian attitudes (β = −0.194, *p* < 0.001). The trust in the effectiveness of campaigns (r2), however, did not show a significant association.

This model presents a high explained variance (R^2^ = 0.439), suggesting that these forms of reluctance (r1 and r3) account for a substantial proportion of individual differences in attitudes toward sexual consent, even when controlling for gender and age (see Table 6).

In the case of positive attitudes toward establishing consent (SCS-R F2), the pattern is similar but with differences in magnitude. Here, the trust in the effectiveness of campaigns (r2: β = 0.231, *p* < 0.001) predicted more egalitarian attitudes and the lack of personal identification with the messages (r3: β = −0.107, *p* < 0.001) correlated with less egalitarian ones. Thus, those who distrust the usefulness of campaigns (r2) tend to place less importance on clearly establishing consent. Likewise, those who do not see themselves reflected in institutional messages (r3) also maintain less positive attitudes toward establishing consent. Conversely, the perception of “lecturing” and an exaggerated tone (r1) did not show a significant effect on this dimension. The model explained 21.2% of the variance (R^2^ = 0.212), a noteworthy level for such a specific dimension. Gender did exhibit a significant effect on this dimension (β = 0.241, *p* < 0.05), indicating that women have more egalitarian attitudes, even after controlling for reluctance.

Regarding digital consent, the model shows that all three forms of reluctance are related to the attitudes toward the importance of consent in digital contexts. The perception that campaigns lecture or have an exaggerated tone (r1) presents a significant negative effect (β = −0.139, *p* < 0.001). The trust in the campaigns’ effectiveness (r2) is significantly and positively associated with higher perceived importance of consent in the digital world (β = 0.192, *p* < 0.001). The lack of personal identification (r3) also shows a negative effect, albeit a more moderate one (β = −0.092, *p* < 0.05). Neither gender nor age presented significant effects on attitudes toward digital consent when the three reluctance indicators were included.

Summarizing these results and answering RQ4, reluctance toward institutional messages predicts more permissive attitudes regarding the absence of consent in both sexual and digital domains. The perception that campaigns exaggerate is the most consistent predictor, affecting both GASC and digital consent. In turn, a lack of trust in the effectiveness of campaigns is especially associated with less positive attitudes toward establishing consent (SCS-F2) and toward digital consent. Finally, lack of personal identification with the messages shows a cross-cutting effect on all variables, although of moderate magnitude.

These models indicate that the different forms of institutional reluctance operate as relevant predictors, albeit with moderate effect sizes, of attitudes toward sexual and digital consent. When r1, r2, and r3 are included in the models, the explained variance increases beyond that accounted for by sociodemographic factors alone, reinforcing the idea that how institutional messages are perceived and evaluated is a key element in understanding attitudinal positions regarding consent.

## 5. Discussion

The aim of this study was to analyze the relationship between attitudes toward sexual consent and consent in digital contexts, as well as to examine the role of reluctance toward institutional messages as a possible moderator of these attitudes. In addition, the study sought to identify which sociodemographic factors are associated with more egalitarian attitudes toward consent and with higher levels of skepticism toward public campaigns. These questions were formulated in line with the primary prevention framework of sexual violence presented in the introduction. This framework focused on modifying risk and protective factors at multiple levels [3,4]. The questions were also formulated according to the conceptualization of sexual consent as a multidimensional and relational construct developed in the theoretical section [22,23]. The results allow for an integrated discussion of three axes: (1) The continuity between sexual and digital consent, (2) The structuring role of gender, and (3) The function of institutional reluctance and its implications for the design of campaigns and policies.

### 5.1. Continuity Between Sexual Consent and Digital Consent

Regarding RQ1, the results show a clear association between the global index of attitudes toward sexual consent (GASC) and the digital consent index. This finding provides empirical support for the idea set out in the theoretical framework that consent is not a one-off act but a relational and contextual process that integrates an internal (desire, comfort, safety) and an external dimension (verbal and nonverbal forms of expression) (Hickman & Muehlenhard, 1999; Jozkowski & Peterson, 2013; [23,46]). Far from there being “two moral codes”—one for the physical and another for the digital world—the data point to a shared normative repertoire: those who tend to value explicit consent in offline interactions also regard it as essential when it comes to sending or sharing sexual content online.

This result confirms and adds nuance to the literature on digital consent discussed in the introduction. Studies such as those by Ringrose et al. (2013, 2023) [13,14] and Powell & Henry (2017) [12] have stressed that the circulation of intimate images and revenge porn introduces specific vulnerabilities (permanence, uncontrollable reach, irreversibility, new forms of abuse), which could give rise to differentiated norms for the online domain. Without denying these particularities, our data suggest that digital consent functions less as an autonomous domain and more as an extension of the same ethical framework that structures consent in face-to-face interactions. While this continuity does not imply absolute equivalence between contexts, it does indicate a normative coherence that is crucial for the design of preventive interventions.

The clearest contribution of this study at this point is showing that this continuity is articulated through specific dimensions of consent. As discussed in the section on the SCS-R, the subscales Positive attitude toward establishing consent (SCS-F2) and (Lack of) perceived behavioral control (SCS-F1) are directly inspired by the Theory of Planned Behavior [32,33]. In line with that framework, our results indicate that the combination of favorable attitudes and high self-efficacy is a strong predictor of digital consent: those who view requesting and negotiating consent positively and perceive themselves as capable of doing so are also those who most strongly reject sending or disseminating sexual content without permission.

The study therefore provides evidence to refine the primary prevention models reviewed in the introduction [3,4]: it is not enough to incorporate “the digital” as a new risk context; interventions must work on attitudes toward consent as an everyday practice (not only as a legal requirement) and on perceived behavioral control. Accordingly, consent-negotiation skills are perceived as transferable between offline and online contexts. This finding is consistent with recent proposals that emphasize how to request and respect consent rather than simply repeating abstract normative slogans [5]. However, our data also warn that these approaches are limited when defensive responses linked to institutional reluctance arise.

### 5.2. Gender as a Structural Factor in Attitudes Toward Consent

In relation to RQ2, gender emerges as the only sociodemographic factor with clear, consistent, and substantively sized effects across all analyzed dimensions. Specifically, women show more egalitarian attitudes toward sexual consent (i.e., higher scores on the GASC index); stronger positive attitudes toward establishing explicit consent (SCS-F2); and lower perceived difficulty in negotiating consent (SCS-F1). Furthermore, they assign greater importance to digital consent and report a lower perceived lack of behavioral control. This empirical finding reaffirms the centrality of gender in the construction of consent, as discussed in frameworks based on sexual script theory [28] and on hegemonic masculinity [47].

In terms of cultural scripts, our results are consistent with the idea that men continue to be socialized, to a greater extent, as legitimate initiators of sexual contact, while women are still assuming responsibility for “managing” risk and regulating sexual access [48,49]. The fact that women display greater adherence to explicit consent and higher perception of control can be interpreted in two ways: first, as an indication of the internalization of egalitarian frameworks promoted by equality policies and recent feminist campaigns; and second, as a response to the accumulated experiences of risk and vulnerability to offline and digital sexual violence [16].

Analytically, our findings allow us to move beyond a mere statement of gender differences. On the one hand, they show that, even when controlling for age, income, educational level, social media use, and relationship status, gender remains the main structuring axis of attitudes. This pattern is consistent with theoretical frameworks that locate the roots of sexual violence in structural gender inequalities rather than exclusively in individual information deficits [28,35,47]. However, as the present data measure attitudes rather than direct adherence to gender norms or sexist beliefs, they do not allow for a definitive causal claim. Moreover, the absence of significant interactions between gender and other sociodemographic variables reinforces the idea that it operates as a determining axis in this analytical context. On the other hand, by extending these differences to the realm of digital consent, the study confirms the theses in the literature that describe technology-mediated violence as a continuation of gender-based violence on new platforms, rather than as a completely new phenomenon [12,15].

Finally, the lack of systematic associations with other sociodemographic variables opens a line of intersectional reflection: our data suggest that, in this sample and in the specific context of a municipal campaign, gender acts as the dominant axis. However, future research should explore how gender intersects with other factors (class, origin, sexual orientation, age) in shaping consent, as intersectional approaches to gender-based violence and digital citizenship have advocated.

### 5.3. Institutional Reluctance as a Predictor of Less Egalitarian Attitudes

The third contribution of the study lies at the intersection of RQ3 and RQ4 as well as the framework on institutional trust, reactance, and unintended campaign effects presented in the introduction [7,8,10]. Although the term institutional reluctance is not yet a consolidated theoretical concept in the literature, we have proposed operationalizing it through three dimensions: perception of a moralizing or exaggerated tone (r1), trust in the effectiveness of campaigns (r2), and lack of personal identification with the campaign’s messages (r3). The results indicate that these three forms of reluctance are associated with less egalitarian attitudes toward sexual and digital consent, albeit with partially differentiated patterns.

From the perspective of psychological reactance [7], the association between r1 and less egalitarian attitudes—here, lower endorsement of explicit consent norms as measured by GASC and the digital consent index—can be interpreted as an example of a boomerang effect. When institutional messages are perceived as moralizing sermons, defensive mechanisms are activated that lead to reaffirmation of prior positions and rejection of the proposed norm [8,9]. Our data do not allow causal claims, but they do show that those who describe campaigns as “exaggerated” are systematically less favorable to consent, both in sexual and digital domains. Importantly, the cross-sectional design does not allow us to determine whether reluctance preceded exposure to the campaign or was triggered by it. It is plausible either that individuals with pre-existing less favorable attitudes toward consent perceived the campaign as exaggerated, or conversely, that the campaign itself—through its tone or framing—generated the reluctance observed. This temporal ambiguity is a recognized limitation of cross-sectional survey designs in campaign evaluation research [8], warranting further longitudinal research to address these causal pathways. This supports existing criticisms in the literature of campaigns based on unidirectional messages that are ill-suited to people’s real experiences. This pattern aligns with preventive communication models, which show that when risk is attributed to “others,” willingness to reconsider one’s own practices decreases.

In the case of r2, trust in the effectiveness of campaigns connects with the literature on institutional legitimacy. Following Tyler [10], the acceptance of institutional norms and decisions depends largely on whether people perceive institutions as fair, consistent, and effective. In our study, those who doubt that campaigns are useful for preventing assaults tend to show less favorable attitudes toward the explicit establishment of consent and the importance of digital consent. This association suggests that a lack of confidence in the transformative capacity of institutions not only affects support for policies in the abstract, but also shapes how the normative content of campaigns is evaluated.

Finally, lack of identification with the messages (r3) contributes to debates on segmentation and perceived relevance in health and prevention communication [3]. When people consider that messages “are not for me,” they are more likely to locate the problem in “others” and to maintain their own practices and attitudes. This result underscores the importance of developing segmented, culturally relevant messages that connect with diverse experiences and trajectories rather than addressing an abstract audience.

### 5.4. Implications for Primary Prevention and Social Policies

From a public policy perspective, the findings of this study directly relate to the primary prevention framework presented in the introduction [3,4] and to recent proposals on consent campaigns [5]. Three main implications can be highlighted.

First, the continuity between sexual and digital consent supports proposals advocating the integration of sexual ethics and digital citizenship within a single intervention architecture. Models that sharply separate “sexual violence” and “digital violence” risk fragmenting understanding of the problems and duplicating efforts. Our results indicate that working on attitudes and skills related to consent in offline settings—including internal and external consent [22,24]—may indirectly impact how consent is conceived in the exchange of intimate images, provided that the digital dimension is made explicit in educational content and campaigns [12,13]. In this sense, the identified attitudinal continuity suggests that strengthening offline consent skills may yield collateral benefits in digital practices.

Second, the centrality of gender in shaping attitudes and the extension of these differences to the digital field reinforce the structurally gendered nature of sexual and digital violence. This empirically confirms the conclusions of frameworks on gendered sexual scripts and hegemonic masculinity [28,47], which argue that effective prevention requires transforming gender norms that legitimize male dominance and the objectification of women’s bodies rather than relying on ostensibly neutral interventions. In practical terms, this implies designing campaigns and programs that work specifically with men and boys—as key agents of change—and that make visible the connection between hegemonic masculinity, consent, and technology use. Likewise, our results suggest the need to explicitly address male digital socialization, a dimension the literature has identified as critical for preventing risky practices such as forwarding images without consent.

Third, the results on institutional reluctance introduce a dimension that is rarely addressed in classic primary prevention models: the institutional conditions that enable normative change. The literature reviewed in the introduction already pointed to unintended effects of campaigns [8,9] and to the role of institutional legitimacy [10]. Our results provide quantitative evidence that these dynamics are not marginal, but substantively linked to attitudes toward consent. According to them, in order to be consistent with primary prevention principles, policies must combine:

⋅ More participatory campaigns, co-designed with target groups, that minimize moralizing tone and increase identification.
⋅ Institutional reforms that reduce gaps between discourse and practice, for example, by improving responses to reports of offline and digital sexual violence, enhancing access to support resources, or strengthening inter-institutional coordination. In this way, consent messages are perceived as part of a structural commitment rather than as isolated gestures. It is also necessary to strengthen institutional accountability mechanisms so that the public perceives coherence between campaigns and the actual actions of public bodies.

It is important to acknowledge that this study did not evaluate the actual content or quality of the specific municipal campaign, and therefore conclusions about what produced the reluctance observed must be made cautiously. The associations found between perceived tone and less favorable consent attitudes are consistent with reactance theory but do not rule out the possibility that the campaign content itself was a contributing factor.

Even with these constraints, the study contributes three relevant elements to the theoretical and applied debate: (1) Evidence of continuity between sexual and digital consent from a multidimensional perspective, (2) empirical confirmation of the centrality of gender in shaping consent attitudes, and (3) introduction of institutional reluctance as an analytical component for understanding why primary prevention campaigns do not always achieve the expected effects. Integrating these three dimensions into future policies and programs may help bridge the gap between normative frameworks on consent—developed in theory and law—and the everyday practices and experiences of the population, both offline and online.

## 6. Conclusions and Social Policy Directions

This study provides empirical evidence on attitudes toward sexual consent—both in offline and digital contexts—in a sample of adults living in a Spanish city, as well as on the role that reluctance toward institutional messages plays in shaping these attitudes. Several main conclusions can be drawn from the results.

First, the study confirms the existence of clear continuity between attitudes toward both in-person and digital sexual consent. Individuals who give greater importance to explicit consent in sexual encounters also tend to place greater value on the need to obtain it before sending or sharing sexual content online, especially when they show a positive attitude toward establishing consent (SCS-F2). This finding supports the relevance of addressing sexual and digital consent as interrelated dimensions within a single primary prevention framework. Moreover, this continuity is consistent with theoretical models that conceptualize consent as a relational and dynamic process [22,24], suggesting that educational frameworks and campaigns should focus on transferable skills across offline and online contexts rather than on isolated normative messages.

Second, gender emerges as the main sociodemographic axis of differentiation. Women display more favorable attitudes toward the importance of sexual and digital consent, as well as greater perceived confidence in establishing it. By contrast, variables such as age, income, or relationship status do not show consistent effects. This finding reinforces the need for equality policies and prevention strategies to continue challenging traditional sexual scripts and to work specifically with men, promoting models of masculinity that incorporate consent as an ordinary and desirable practice. In light of the reviewed literature, this pattern confirms that gender inequalities continue to structure the contexts in which consent is negotiated [28,35], underlining that transforming attitudes cannot be separated from transforming social norms and cultural expectations about gender roles.

Third, institutional reluctance emerges as a key factor in explaining less egalitarian attitudes toward consent. The perception that institutional campaigns exaggerate or adopt a moralizing tone, distrust in their effectiveness, and lack of personal identification with their messages are differentially associated with a lower valuation of sexual and digital consent. These results indicate that producing more campaigns or merely refining their normative content is not enough. It is also essential to address how these messages are perceived and received, as well as the relationships of trust (or mistrust) between citizens and institutions. This finding connects with the literature on reactance and unintended effects in preventive communication [7,8], reinforcing the idea that campaigns may lose effectiveness—or even generate rejection—when they are not perceived as legitimate, relevant, or connected to the everyday realities of their audiences.

From a social policy perspective, the findings highlight several concrete directions:

⋅ Systematically integrate digital consent into primary prevention strategies, linking it to affective-sexual education and critical digital literacy, so that offline and online dimensions of consent are addressed jointly.
⋅ Design campaigns and programs that challenge unequal gender scripts, with particular attention to male socialization. This implies combining messages aimed at young and adult men with deeper spaces for reflection, in coordination with educational institutions, community organizations, and social movements.
⋅ Review the tone and formats of institutional campaigns on consent, avoiding moralizing or overly simplistic approaches. It is crucial to move toward co-design and co-creation models with target groups, allowing language, images, and examples to be adapted to their real experiences and, at the same time, helping rebuild trust in institutions.
⋅ Embed campaigns within broader structural change strategies, including reforms in education, health, justice, and social services systems, so that messages about consent do not appear as isolated communicative “islands” disconnected from everyday institutional practices. In this sense, the articulation between communication and institutional coherence—highlighted in the literature on institutional legitimacy [10]—is essential for preventive messages to be perceived as part of an ongoing commitment rather than as one-off interventions.

This study is not without limitations that must be considered. The sample, although stratified by age and gender, is local and non-probabilistic, so the possibilities for generalization are necessarily partial. The data are cross-sectional and based on self-reports, which prevents the establishment of firm causal relationships and may introduce social desirability biases. In particular, the cross-sectional design does not allow us to determine the temporal direction of the association between institutional reluctance and consent attitudes: it remains unknown whether reluctance preceded campaign exposure or was triggered by it. Additionally, this study did not evaluate the actual content or quality of the campaign messages. Consequently, it is not possible to rule out that the content itself contributed to the reluctance observed, independently of its institutional source. In addition, digital consent was measured with only two items, and institutional reluctance was assessed through three indicators that do not form a unidimensional scale, suggesting the need to continue developing and validating specific instruments in this field. Complementarily, recent literature highlights the need to incorporate qualitative methods that can capture the affective and narrative dimensions of consent and institutional mistrust [14], which can hardly be covered by brief instruments.

Future research could deepen the analysis of the relationship between attitudes and actual behaviors in offline and digital contexts, explore the evolution of institutional reluctance over time, and examine intersectional differences (by age, social class, ethnic background, or nationality) that this study could not address in detail. It would also be particularly relevant to combine quantitative and qualitative approaches to better understand how experiences of consent and institutional mistrust are constructed in everyday life. In addition, it would be pertinent to analyze whether the repetition or continuity of campaigns—beyond one-off exposure—modulates levels of reluctance and promotes the consolidation of egalitarian norms, a question that international evidence has not yet systematically addressed.

Despite these limitations, the results offer valuable information for the design of equality policies and primary prevention strategies for sexual violence. By showing the continuity between sexual and digital consent, the centrality of gender, and the relevance of institutional reluctance, the study underscores the importance of comprehensive policies that combine normative change, transformations in gender scripts, and the building of trust between citizens and institutions. Taken together, the findings reinforce the idea that effective prevention of sexual violence requires simultaneous intervention in individual practices, cultural norms, and institutional structures, avoiding approaches that are exclusively informative or focused on correcting isolated behaviors.

Advancing toward societies where consent is an integral part of everyday practice requires coherent public policies, ongoing research, and an informed and empowered citizenry.

## Figures and Tables

**Table 1:** Sample sociodemographic statistics.

Variable	Category	*n*	%
Gender	Men	175	43.3%
	Women	229	56.7%
Education	Lower Secondary Education/Compulsory Secondary Education	58	14.4%
	Upper Secondary Education (Baccalaureate) or Intermediate Vocational Training	74	18.3%
	Higher Vocational Training	83	20.5%
	University Degree	46	11.4%
	Postgraduate Degree	143	35.4%
Social media use	Never or almost never	33	8.2%
	Occasionally	17	4.2%
	Two to three times per week	30	7.4%
	Four to five days per week	22	5.4%
	Every day or almost every day	302	74.8%
Household income	Less than €1000 per month	20	5.0%
	€1000 to €2000 per month	134	33.2%
	€2000 to €3000 per month	105	26.0%
	€3000 to €4000 per month	73	18.1%
	€4000 to €5000 per month	30	7.4%
	More than €5000 per month	16	4.0%
	na	26	6.4%
Relationship status	Partner	287	71.0%
	No partner	110	27.2%
	na	7	1.7%
Age	Mean	42.3	42.3
	sd	12.9	12.9

na: not answered; sd: standard deviation.

**Table 2:** SCS-R items used.

Item Original Number	Items Used in: Rollero et al. [34].	Translation into Spanish by: Moyano et al. [26]
D2 (Lack of) perceived behavioral control (i.e., how much behavioral control over sexual consent negotiations do participants perceive)		
Item 5	I think that verbally asking for sexual consent is awkward.	Pienso que pedir consentimiento sexual de forma verbal es incómodo
Item 6	I have not asked for sexual consent (or given my consent) at times because I felt that it might backfire and I wouldn’t end up having sex.	No he pedido consentimiento sexual (ni he dado mi consentimiento) algunas veces porque pensé que entonces no tendría relaciones sexuales
Item 7	I believe that verbally asking for sexual consent reduces the pleasure of the encounter.	Creo que pedir consentimiento sexual de forma verbal reduce el placer del encuentro
D2 Positive attitude toward establishing consent		
Item 14	I think it is equally important to obtain sexual consent in all relationships regardless of whether or not they have had sex before.	Pienso que es importante obtener el consentimiento sexual en todas las relaciones sexuales, indistintamente de si se han tenido o no relaciones sexuales con esa persona
Item 17	I believe that it is just as necessary to obtain consent for genital fondling as it is for sexual intercourse.	Creo que es tan necesario obtener consentimiento para caricias genitales como para las relaciones sexuales coitales
Item 20	I feel it is the responsibility of both partners to make sure sexual consent is established before sexual activity begins.	Opino que es responsabilidad de ambos miembros de la pareja asegurarse de que se establezca el consentimiento sexual antes de que comience la actividad sexual
D3 Indirect behavioral approach to consent (i.e., the use of indirect, nonverbal ways to negotiate sexual consent)		
Item 25	Typically I ask for consent by making a sexual advance and waiting for a reaction, so I know whether or not to continue.	Por lo general, pido consentimiento haciendo un acercamiento sexual y esperando una reacción, para saber si continuar o no
Item 26	I don’t have to ask or give my partner sexual consent because my partner knows me well enough.	No tengo que pedir ni dar mi consentimiento sexual a mi pareja porque mi pareja me conoce suficientemente bien
Item 27	I don’t have to ask or give my partner sexual consent because I have a lot of trust in my partner to “do the right thing.”	No tengo que pedir ni dar mi consentimiento sexual a mi pareja porque tengo mucha confianza en que él/ella “hará lo correcto”
D4: Sexual consent norms (i.e., beliefs about the norms that regulate sexual consent negotiations)		
Item 31	I believe that the need for asking for sexual consent decreases as the length of an intimate relationship increases.	Creo que la necesidad de pedir consentimiento sexual disminuye a medida que aumenta la duración de una relación de pareja
Item 32	I believe it is enough to ask for consent at the beginning of a sexual encounter.	Creo que es suficiente pedir el consentimiento al comienzo de un encuentro sexual
Item 35	If consent for sexual intercourse is established, petting and fondling can be assumed.	Si existe consentimiento sexual para relaciones sexuales coitales, se asume que también existe para otras actividades (caricias y tocamientos)
D5 Awareness and discussion		
Item 36	I have discussed sexual consent issues with a friend.	He debatido sobre cuestiones relacionadas con el consentimiento sexual con un amigo/a
Item 38	I have discussed sexual consent issues with my current (or most recent) partner at times other than during sexual encounters.	He debatido sobre cuestiones de consentimiento sexual con mi pareja actual (o la más reciente) en momentos distintos a los encuentros sexuales
Item 39	I have not given much thought to the topic of sexual consent.	No he pensado mucho en el tema del consentimiento sexual

**Table 3:** Regression models (HC2) linking traditional sexual consent attitudes and attitudes towards digital sexual consent.

Predictor	DC~GASC	DC~GASC	DC~GASC (Controlled)	DC~GASC (Controlled)	DC~SCS-F1	DC~SCS-F1	DC~SCS-F1 (Controlled)	DC~SCS-F1 (Controlled)	DC~SCS-F2	DC~SCS-F2	DC~SCS-F2 (Controlled)	DC~SCS-F2 (Controlled)
Intercept	3.122 *** (0.345)	3.122 *** (0.345)	2.636 *** (0.521)	2.636 *** (0.521)	6.562 *** (0.138)	6.562 *** (0.138)	6.082 *** (0.436)	6.082 *** (0.436)	1.437 *** (0.295)	1.437 *** (0.295)	1.176 ** (0.384)	1.176 ** (0.384)
GASC	0.593 *** (0.070)	0.593 *** (0.070)	0.577 *** (0.072)	0.577 *** (0.072)	—	—	—	—	—	—	—	—
SCS-F1	—	—	—	—	−0.254 *** (0.043)	−0.254 *** (0.043)	−0.235 *** (0.044)	−0.235 *** (0.044)	—	—	—	—
SCS-F2	—	—	—	—	—	—	—	0.740 *** (0.047)	0.740 *** (0.047)	0.732 *** (0.048)	0.732 *** (0.048)	
Gender (Woman)	—	0.319 * (0.137)	0.319 * (0.137)	—	—	0.333 * (0.147)	0.333 * (0.147)	—	—	0.152 (0.110)	0.152 (0.110)	
Age	—	0.008 (0.005)	0.008 (0.005)	—	—	0.003 (0.005)	0.003 (0.005)	—	—	0.003 (0.004)	0.003 (0.004)	
Education (ordinal)	—	0.098 (0.053)	0.098 (0.053)	—	—	0.105 (0.054)	0.105 (0.054)	—	—	0.035 (0.042)	0.035 (0.042)	
Social media use (ordinal)	—	−0.071 (0.051)	−0.071 (0.051)	—	—	−0.066 (0.052)	−0.066 (0.052)	—	—	−0.027 (0.038)	−0.027 (0.038)	
Income (ordinal)	—	0.024 (0.069)	0.024 (0.069)	—	—	0.027 (0.073)	0.027 (0.073)	—	—	0.041 (0.044)	0.041 (0.044)	
Relationship status (no partner)	—	−0.139 (0.172)	−0.139 (0.172)	—	—	−0.145 (0.181)	−0.145 (0.181)	—	—	−0.036 (0.125)	−0.036 (0.125)	
N	404	371 ^1^	371	404	404	371	371	404	404	371	371	
R^2^	0.141	0.184	0.184	0.085	0.085	0.122	0.122	0.430	0.430	0.479	0.479	
RMSE	1.33	1.28	1.28	1.37	1.37	1.32	1.32	1.08	1.08	1.02	1.02	

* *p* < 0.05, ** *p* < 0.01, *** *p* < 0.001. DC = digital consent. ^1^ When sociodemographic factors are included, the sample size decreases to 371 due to missing data in the income variable.

**Table 4:** Regression models linking attitudes towards sexual consent and sociodemographic factors.

Predictor	GASC	Digital Consent	SCS-F1	SCS-F2
Constant	4.436 *** (0.298)	5.194 *** (0.431)	3.777 *** (0.560)	5.485 *** (0.395)
Gender (Women)	0.240 * (0.097)	0.458 ** (0.149)	−0.531 ** (0.173)	0.417 ** (0.137)
Age	−0.006 (0.004)	0.005 (0.006)	−0.005 (0.007)	0.002 (0.005)
Education (ordinal)	0.035 (0.036)	0.118 * (0.056)	−0.053 (0.065)	0.112 * (0.050)
Social media use (ordinal)	0.011 (0.040)	−0.064 (0.054)	−0.005 (0.078)	−0.051 (0.051)
Income (ordinal)	0.011 (0.045)	0.030 (0.075)	−0.011 (0.080)	−0.015 (0.065)
Relationship status (no partner)	0.015 (0.108)	−0.130 (0.184)	−0.063 (0.193)	−0.128 (0.166)
N	371	371	371	371
R^2^	0.030	0.051	0.031	0.046
RMSE	0.90	1.38	1.61	1.26

Standard errors in parentheses. * *p* < 0.05, ** *p* < 0.01, *** *p* < 0.001.

**Table 5:** Regression models linking reluctance and sociodemographic factors.

Predictor	R1	R2	R3
Interceptor	5.542 *** (0.637)	4.037 *** (0.553)	3.403 *** (0.678)
Gender (Women)	−0.768 *** (0.197)	0.344 (0.191)	−0.114 (0.212)
Age	−0.018 * (0.008)	0.009 (0.008)	0.008 (0.009)
Education (ordinal)	−0.126 (0.073)	0.036 (0.068)	−0.119 (0.077)
Social media use (ordinal)	−0.137 (0.085)	0.083 (0.073)	0.016 (0.089)
Income (ordinal)	−0.031 (0.093)	−0.042 (0.092)	0.021 (0.102)
Relationship status (no partner)	−0.048 (0.234)	−0.127 (0.238)	−0.046 (0.243)
N	371	371	371
R^2^	0.076	0.019	0.010
RMSE	1.82	1.79	1.97

Standard errors in parentheses.* *p* < 0.05, *** *p* < 0.001.

**Table 6:** Regression models linking reluctance and attitudes towards sexual consent.

Predictor	GASC	SCS-F2	Digital Consent
Interceptor	5.841 *** (0.205)	5.035 *** (0.359)	5.151 *** (0.389)
R1	−0.164 *** (0.025)	−0.063 (0.035)	−0.139 *** (0.041)
R2	0.028 (0.022)	0.231 *** (0.039)	0.192 *** (0.045)
R3	−0.194 *** (0.023)	−0.107 *** (0.028)	−0.092 * (0.037)
Gender (Women)	0.066 (0.071)	0.241 * (0.119)	0.181 (0.137)
Age	−0.008 ** (0.003)	0.003 (0.004)	0.008 (0.005)
N	404	404	404
R^2^	0.439	0.212	0.183
RMSE	0.68	1.13	1.30

Standard errors in parentheses. * *p* < 0.05, ** *p* < 0.01, *** *p* < 0.001.

## Data Availability

The data supporting the findings of this study are available from the corresponding author upon reasonable request.
